# Aryl Hydrocarbon Receptor Promotes Cell Growth, Stemness Like Characteristics, and Metastasis in Human Ovarian Cancer via Activation of PI3K/Akt, β-Catenin, and Epithelial to Mesenchymal Transition Pathways

**DOI:** 10.3390/ijms23126395

**Published:** 2022-06-07

**Authors:** Lubna Therachiyil, Roopesh Krishnankutty, Fareed Ahmad, Jericha M. Mateo, Shahab Uddin, Hesham M. Korashy

**Affiliations:** 1 Department of Pharmaceutical Sciences, College of Pharmacy, QU Health, Qatar University, Doha P.O. Box 2713, Qatar; lt1904995@qu.edu.qa; 2 Translational Research Institute, Academic Health System, Hamad Medical Corporation, Doha P.O. Box 2713, Qatar; rkrishnankutty@hamad.qa (R.K.); fahmad8@hamad.qa (F.A.); jmateo1@hamad.qa (J.M.M.); skhan34@hamad.qa (S.U.); 3 Dermatology Institute, Academic Health System, Hamad Medical Corporation, Doha P.O. Box 2713, Qatar

**Keywords:** ovarian cancer, aryl hydrocarbon receptor, chemoresistance, EMT, apoptosis, stemness

## Abstract

Ovarian cancer (OC) ranks first in cancer-related deaths out of all female reproductive malignancies with high-pitched tumor relapse and chemoresistance. Several reports correlate cancer occurrences with exposure to xenobiotics via induction of a protein receptor named aryl hydrocarbon receptor (AhR). However, the effect of AhR on OC proliferation, expansion, and chemoresistance remains unrevealed. For this purpose, OC cells A2780 and A2780cis cells were treated with AhR activator, 2,3,7,8-tetrachlorodibenzo-p-dioxin (TCDD), and the effects were determined by Real-Time Cell Analyzer, clonogenic assay, flow cytometry, immunoblotting and wound healing assay. Our results showed that activation of AhR by TCDD in A2780 cells induced the PI3K/AKT pathway followed by induction of anti-apoptotic proteins BCL-2, BCL-xl, and MCL-1. In addition, a significant increase in stemness marker aldehyde dehydrogenase (ALDH1) was observed. This effect was also associated with an accumulation of β-catenin, a Wnt transcription factor. Moreover, we observed induction of epithelial to mesenchymal transition (EMT) upon AhR activation. In conclusion, the results from the current study confirm that AhR mediates OC progression, stemness characteristics, and metastatic potential via activation of PI3K/Akt, Wnt/β-catenin, and EMT. This study provides a better insight into the modulatory role of AhR that might help in developing novel therapeutic strategies for OC treatment.

## 1. Introduction

Ovarian cancer (OC), globally the most lethal gynecological malignancy, ranks fifth in terms of cancer-related deaths [1]. Regardless of an efficient treatment regimen, including cytoreductive surgery followed by chemotherapy, OC exhibits high tumor relapse and chemoresistance [2]. Unfortunately, Stage IV OC’s relative five-year survival rate is only 17%, which can be highly correlated to chemoresistance [3]. The major risk factors associated with OC include genetic mutations [4], family history, reproductive factors, lifestyle [5], and environmental factors [6]. Previous reports have related exposure to xenobiotics to cancer initiation [7,8]. Reports suggest that a major pathway that regulates xenobiotic detoxification is the aryl hydrocarbon receptor (AhR) pathway, whose involvement in several cancer and cancer stem cells (CSCs) was recently demonstrated [9]. 

AhR is a cytoplasmic ligand-activated transcriptional factor associated with several cellular and physiological processes in the cell [10]. Upon binding to its ligands, such as 2,3,7,8-tetrachlorodibenzo-p-dioxin (TCDD), AhR transcriptionally activates several genes, including the cytochrome P4501A1 (CYP1A1) and CYP1B1 involved in the bioactivation of several environmental procarcinogens into ultimate carcinogens [7]. A correlation between the AhR expression and cancer initiation and its potential use as a therapeutic target has been explained by several researchers in breast [11], glioblastoma [12], gastric [13], and lymphoma [14], colon [15], and melanoma [16]. In OC, a previous study has evidenced that OC patients with lower AhR expression in the cytoplasm have a better prognosis [17]. However, its role in ovarian cancer development and chemoresistance remains uninvestigated.

Therefore, in our study, we examined the role and involvement of AhR in OC progression and chemoresistance and the underlying mechanisms of AhR in the pathogenesis of ovarian cancer. We observed that AhR induction by a potent activator TCDD promoted cell proliferation in A2780 cells by activating the PI3K/AKT pathway. We also observed an upregulation of anti-apoptotic and stemness markers further confirming AhR mediated induction of chemoresistance (by evading apoptosis) and stemness-like characteristics. Moreover, AhR induction also promoted EMT in A2780 cells. The results from our study provide a better insight into the modulatory role of AhR that might help in developing novel therapeutic strategies for OC treatment.

## 2. Results 

### 2.1. Constitutive and Inducible Expression of AhR and BCL Family Proteins in OC Cells

#### 2.1.1. Basal Expression of AhR and BCL-2 Proteins in OC Cell Lines

**Figure 1:**
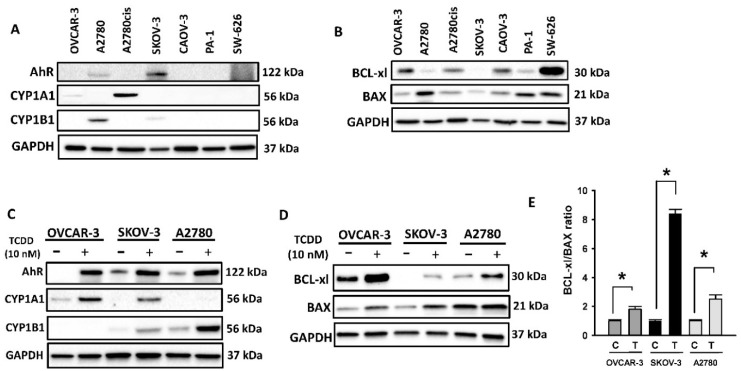
Constitutive and inducible expression of AhR and BCL family proteins in OC cells. Immunoblot analysis of (**A**,**B**) constitutive and (**C**,**D**) TCDD-inducible protein expression levels of the target genes in OC cells using GAPDH as a housekeeping gene. The protein panels are a composite of different blots and are representative blots for the indicated proteins and the loading control. (**E**) Fold change of Bcl-xl to BAX ratio in constitutive and TCDD-inducible OC. Intensities of each bad were quantified using ImageJ software mean ±SD (*n* = 3). *; *p* < 0.05 compared to the corresponding control.

We first identified the basal expression of AhR and its regulatory proteins, CYP1A1 and CYP1B1, in all seven human OC cells. Figure 1A shows that all OC cells exhibited a differential expression, in which SKOV-3 cells exhibited the highest basal expression of AhR, while A2780cis and A2780 expressed the highest basal expression of CYP1A1 and CYP1B1 proteins, respectively. We further linked the expression of AhR and its genes with the levels of chemoresistance-mediated BCL-2 family proteins. Figure 1B shows that the basal expression of Bax (pro-apoptotic) was the highest in A2780 cells, whereas SW626 expressed the highest level of BCL-xl (anti-apoptotic) protein. 

#### 2.1.2. TCDD-Inducible Expression of AhR and BCL-2 Proteins in OC Cells

The TCDD-inducible expression levels of AhR and BCL-2 proteins were analyzed by immunoblotting in three OC OVCAR3, SKOV3, and A2780 cells, which showed the highest basal expression, treated with TCDD (10 nM, 24 h). Figure 1C shows that AhR inducer TCDD significantly induced the protein level of AhR in all cells, CYP1A1 in OVCAR-3 and SKOV-3 cells, and CYP1B1 in SKOV-3 and A2780 cells. Importantly, a similar pattern of expression was also observed for BCL-2 proteins, BCL-xl, and BAX (Figure 1D). Although TCDD induced both BAX, a pro-apoptotic protein, and BCL-xl, an anti-apoptotic protein, the ratio of Bcl-xl to Bax that showcases the apoptosis hindrance rate was higher in all three cell lines (Figure 1E). 

### 2.2. Effect of AhR Activation on the Chemoresistance of OC

#### 2.2.1. Effect of AhR Activation on Cell Viability and Clonogenicity of Wild-Type and Resistant A2780 Cells

**Figure 2:**
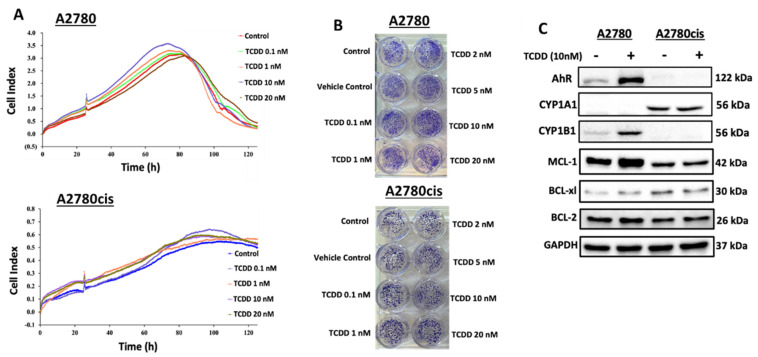
Effect of AhR activation on cell growth, cell survival, and AhR and BCL-2 family proteins in A2780 and A2780cis cell lines. A2780 and A2780cis cells were treated with wide concentrations of TCDD at different time intervals. (**A**) Cell growth was real-time monitored for 120 h. (**B**) Cell survival was determined by colony formation assay analysis. Protein expression levels of (**C**) AhR, CYP1A1, CYP1B1, MCL−1, BCL−2, and BCL−xl proteins in response to 24 h TCDD treatment were determined by Western blot analysis. The protein panels are a composite of different blots and are representative blots for the indicated proteins and the loading control.

Initially, we examined the impact of AhR induction using TCDD on the cell viability of both wild-type A2780 and resistant A2780cis cells. For this purpose, both cells were treated with wide doses of TCDD (0.1, 1, 2, 5, 10, and 20 nM); thereafter, RTCA analysis and clonogenic assay were conducted. RTCA analysis (Figure 2A) and clonogenic assay (Figure 2B) showed that all tested concentrations significantly increased cell viability and clonogenicity of both cells in a dose- as well as time-dependent manner. While the peak cell viability was achieved at 80 h and then decreased to normal at 120 h for A2780, A20780cis cell viability was more resistant and maintained at 120 h. Based on these studies, the TCDD concentration (10 nM) was chosen for all subsequent experiments.

#### 2.2.2. Constitutive and TCDD-Inducible Expression of AhR and BCL-2 Family Proteins in Wild Type and Resistant A2780 Cells

Next, we assessed the effect of AhR activation by TCDD 10 nM for 24 h on the expression patterns of AhR and BCL-2 genes in both wild-type and resistant cells. Interestingly, we observed a differential expression of AhR and its regulatory proteins in A2780 and A2780cis cells. At the basal levels, Figure 2C showed that A2780 cells only express AhR and CYP1B1 but do not express CYP1A1, whereas A2780cis cells expressed only high basal expression of CYP1A1 but no expression for AhR and CYP1B1. At the inducible levels, TCDD treatment markedly induced AhR and CYP1B1 in A2780 cells, while no changes were observed in A2780cis cells. We further explored the expression pattern of BCL-2 family proteins in both cells as illustrated in Figure 2C. While MCL-1, BCL-2, and BCL-xl proteins were expressed at the basal levels in both cells, TCDD (10 nM) treatment induced MCL-1 and BCL-xl in A2780 cells, and did not alter the expression in resistant A2780cis cells. 

#### 2.2.3. Effect of AhR Induction on Apoptosis, Cell Cycle Phases, and DNA Breaks in Wild-Type and Resistant A2780 Cells

**Figure 3:**
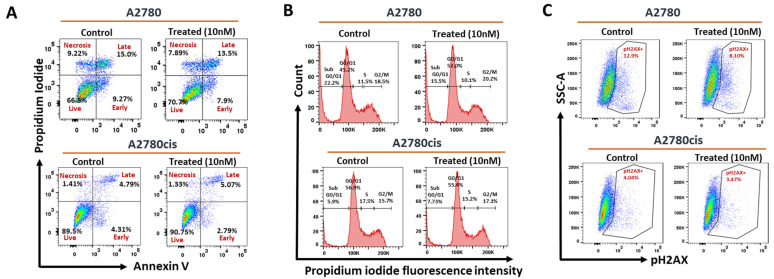
Effect of AhR activation on apoptosis, cell cycle, and phoshoH2AX levels in A2780 and A2780cis cell lines. A2780 and A2780cis cells were treated with wide concentrations of TCDD at different time intervals. (**A**) apoptosis, (**B**) cell cycle phases, and (**C**) phoshoH2AX levels in both A2780 and A2780cis cells were determined by flow cytometric analysis.

To explore the mechanisms of AhR-mediated chemoresistance in OC, we investigated TCDD-mediated effects in modulating apoptosis, cell cycle phases, and level of DNA damage in n both cells. Flow cytometric analysis revealed that treatment of A2780 cells with TCDD 10 nM decreased the percentage of cells undergoing early/late apoptosis to 21.4% (Figure 3A) and increased the percentage of cells in the SubG0-G1 (Figure 3B), while decreased expression levels of DNA double-strand breaks (DSBs) marker, phosphoH2AX (Figure 3C). Interestingly, TCDD treatment of resistant A2780cis cells did not alter the levels of apoptosis, cell cycle, and DNA break (Figure 3A–C).

### 2.3. Molecular Mechanisms Regulating the Effect and Involvement of AhR in OC Chemoresistance

**Figure 4:**
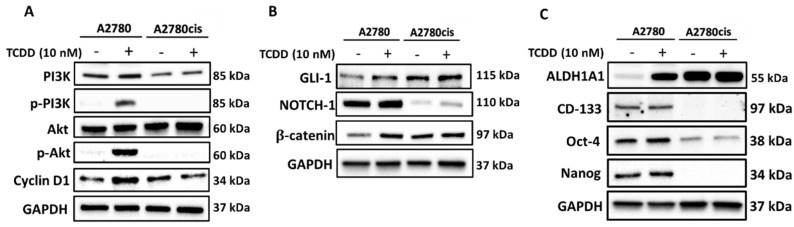
Effect of AhR activation on transcription factors and stemness markers in A2780 and A2780cis cells. A2780 and A2780cis cells were treated for 24 h with 10 nM TCDD. Protein expression levels of (**A**) PI3K, p−PI3K, Akt, p-Akt, and Cyclin D1, (**B**) GLI−1, NOTCH1, and β−catenin, (**C**) ALDH1A1, CD−133, Oct−4, and Nanog were determined by Western blot analysis. The protein panels are a composite of different blots and are representative blots for the indicated proteins and the loading control. The semi−quantitative analysis of the expression of the target proteins is presented in Figure S1.

To investigate whether AhR induction would regulate the PI3K/Akt/Cyclin D1 pathway, we measured the protein expression of PI3K, AKT, and cyclin D1 in both A2780 and resistant A2780cis cells. As shown in Figure 4A, both cells showed a basal expression of these proteins, whereas TCDD treatment induced the expression of phosphorylated p-PI3K, p-AKT, and cyclin D1. On the other hand, treatment of resistant A2780cis cells with TCDD did not induce any of these genes. 

To further explore whether AhR is mediating CSCs properties, self-renewal, and maintenance, in OC A2780 and A2780cis cells, we examined the expression of three proteins, GLI-1, NOTCH1, and β-catenin, the downstream proteins for Hedgehog, NOTCH, and WNT pathways, respectively [18,19]. Figure 4B shows that while the three proteins were expressed in both cells at the basal levels, TCDD treatment only induced β-catenin levels in wild-type A2780 cells and slightly induced NOTCH1, but not the others in resistant A2780cis cells. To further validate these results, we examined the expression of several stemness markers, ALDH1, CD133, OCT4, and NANOG in both cells in response to TCDD. At the basal levels, all these proteins are expressed in A2780 cells, while only ALDH1 and Oct4 are expressed in resistant cells. At the inducible levels, AhR activation by TCDD increased the expression of ALDH1A1 in A2780 cells but in resistant A2780cis cells (Figure 4C). 

**Figure 5:**
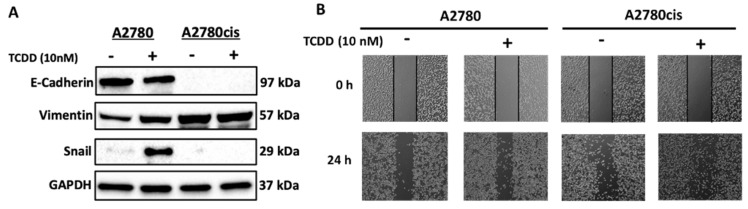
Effect of AhR activation on EMT levels and metastasis potential in A2780 and A2780cis cells. A2780 and A2780cis cells were treated for 24 h with 10 nM TCDD. (**A**) Protein expression levels of E−cadherin, vimentin, and snail were determined by Western blot analysis. The protein panels are a composite of different blots and are representative blots for the indicated proteins and the loading control. (**B**) Migratory behavior was performed using a wound−healing assay. The semi−quantitative analysis of the expression of the target proteins is presented in Figure S2.

We then inspected the involvement of EMT and invasion in response to AhR activation by measuring the epithelial/mesenchymal markers and the metastasis potential. As illustrated in Figure 5A, resistant cells express higher mesenchymal marker vimentin as compared to A2780 cells. Importantly, AhR activation by TCDD in A2780 cells decreased E-cadherin, an epithelial marker, while increased vimentin and snail, major mesenchymal markers. Moreover, TCDD treatment in resistant cells did not alter the expression of any of these proteins (Figure 5A). Furthermore, cell migration of both the cell lines was analyzed by scratch assay, which showed that TCDD increased the rate of wound closure in both cells, which was more observed in resistant A2780cis than A2780 cells (Figure 5B).

## 3. Discussion

Despite the fact that most patients respond to primary therapy in OC, a higher rate of relapse due to chemoresistance is unfortunately observed at a surprising 70% occurrence rate in OC, leading to an inevitable death rate of 90% of patients with relapse [20]. Environmental factors, such as exposure to certain pollutants, such as TCDD, play a major role in the initiation and succession of OC [21]. Given that TCDD and other environmental pollutants mediate their carcinogenic effect through the activation of AhR, there is a lack of information about the role of AhR in OC progression and chemoresistance. In our study, we intended to study the role of AhR in OC progression and chemoresistance and explore the underlying mechanisms. 

Seven human OC cell lines have been utilized in the current study to initially investigate and correlate the expression patent of AhR and regulated genes with the aggressiveness of cancer. Among these cells, OVCAR3, SKOV3, and A2780 cells exhibited higher protein expression levels of AhR, CYP1A1, and CYP1B1, which was associated with a proportional increase in the expression of anti-apoptotic proteins, BCL-2, BCL-xl, and MCL-1. To the best of our knowledge, this is the first report that describes the crosstalk between AhR and BCL-2 in OC and proposes a role of the AhR/CYP1 pathway in OC chemoresistance. The anti-apoptotic role of AhR was previously reported in breast cancer [22], UVB-irradiated human keratinocytes [23], and primary rat hepatocytes by inhibiting pro-apoptotic proteins [24]. 

The availability of resistant OC cells (A2780cis) allows us to further assess the involvement of AhR in the chemoresistance of OC cells which showed a differential expression of the downstream proteins of AhR and BCL-2 pathways in cisplatin-resistant A2780cis. AhR induction by TCDD in wild-type A2780 cells induced both AhR as well as BCL-2 family proteins, suggesting crosstalk between AhR and BCL-2 in chemoresistance. These results were further confirmed by our observations that AhR induction reduced the proportion of apoptotic OC cells, increased the cells in the G0–G1 phase, and decreased the DNA double-strand breaks. Surprisingly, A2780cis cells were resistant to the anti-apoptotic effect of AhR activation as evidenced by no induction of the levels of BCL-2 proteins, cell cycle phases, and DNA strand break. Although the mechanisms of resistance are not fully understood, this effect could be correlated to the property of drug efflux mechanisms carried out by chemoresistant cell lines [25]. 

Perhaps the most interesting part of the study is exploring the molecular mechanisms modulating the AhR chemoresistance in OC. In this study, we specifically explored three pathways, PI3k/Akt, stemness, and EMT. The PI3K/AKT pathway is one of the most frequently upregulated pathways in several cancers and is found to contribute to cancer cell proliferation, invasion, metastasis, and drug resistance [26,27]. In leukemia, upregulation of the PI3K/AKT pathway is linked to poor prognosis [28]. In this regard, although the PI3K/Akt/cyclin D1 proteins are constitutively expressed in both cells, TCDD only induced these proteins in A2780 but not in resistant cells. This could be attributed to the property of drug efflux mechanisms carried out by chemoresistant cell lines [25]. 

**Figure 6:**
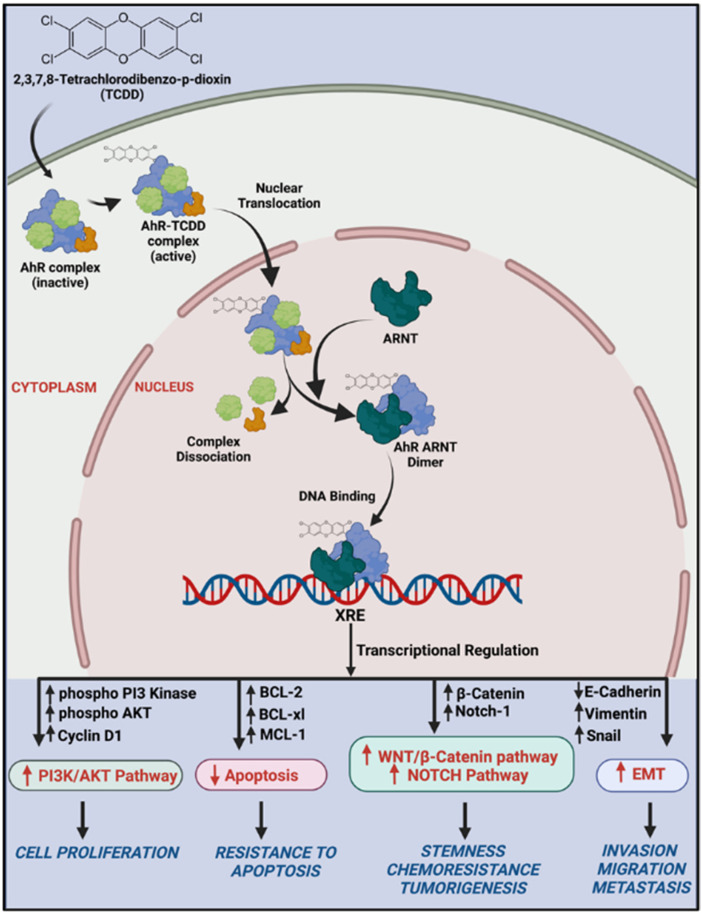
The graphical postulation of the proposed mechanisms (created with Biorender.com).

A previous study in breast cancer MCF-7 cells showed that activation of AhR/CYP1A1 increased the chemoresistance by activating the Wnt/β-catenin and ALDH pathways [8], pathways that mediate CSCs development, maintenance, and self-renewal [29]. Thus, we questioned the involvement of these pathways in AhR-BCL-2 crosstalk in OC. In the current study, we observed that AhR activation induced Wnt/β-catenin, Notch, and ALDH pathways in wild-type A2780 cells, but not in the resistant A2780cis cells, indicating that other pathways might be involved. Studies have shown that activation of β-catenin and Akt pathways by Twist, an important transcriptional factor involved in EMT, is crucial for the maintenance of EMT-associated, CSC-like features in cervical and breast cancer cells [30]. EMT is a highly dynamic biological process, where epithelial cells lose their apical–basal polarity, and their adhesive property and gain a mesenchymal phenotype with enhanced migratory properties, invasiveness, and metastasis [31]. Since our previous results confirmed that the cells were attaining stemness-like properties, and resistance to apoptosis, we questioned whether EMT could be one of the involved mechanisms. We report here, for the first time, that resistant cells express higher levels of mesenchymal markers and have high migration metastatic potentials as compared to wild-type A2780 cells, indicating that EMT could be the main pathway that mediates the chemoresistance of these cells (Figure 6). 

## 4. Materials and Methods

### 4.1. Chemicals and Reagents

TCDD was procured from Toronto Research Chemicals (Toronto, ON, Canada). The antibodies against AhR, CYP1A1, CYP1B1, BCL−2, MCL−1, BCL−xl, and ALDH1A1 were purchased from Santacruz Biotechnology (Heidelberg, Germany), while antibodies against Bax, Oct−4, Nanog, CD−133, E−Cadherin, Vimentin, β−Catenin, PI3K, phospho−PI3K, AKT, phospho−AKT, cyclin D1, GLI−1, NOTCH−1, and GAPDH were bought from Cell Signaling Technologies (Beverly, MA, USA). All kits for flow cytometric analysis were obtained from BD Biosciences (Franklin Lakes, NJ, USA). 

### 4.2. OC Cell Lines and Cell Culture

Human OC cell lines, PA-1 (ATCC CRL-1572TM), OVCAR-3 (ATCC HTB-161TM), CAOV-3 (ATCC HTB-75TM), SKOV-3 (ATCC HTB-77TM), SW 626 (ATCC HTB-78TM) were obtained from the American Type Culture Collection (ATCC, Rockville, MD). A2780 (ECACC 93112519), and cisplatin resistant A2780cis (ECACC 93112519) human OC cells were ordered from European Collection of Authenticated Cell Cultures (ECACC, Salisbury, UK). The cells were grown in Dulbecco’s Modified Eagle’s Medium (DMEM) supplemented with 20% serum (FBS), and 1% antibiotic solution (penicillin 100 mg/mL/streptomycin 10 mg/mL). The cell lines were cultured at 370C in a humidified 5% CO_2_- 95% air atmosphere. 

### 4.3. Protein Extraction and Western Blot Analysis

Protein extraction was carried out as described previously [32]. Fifty micrograms of protein were separated on an SDS polyacrylamide gel followed by immunoblotting as described before [33]. The protein bands were then detected by ECL chemiluminescent substrate (BioRad Western ECL Substrate) and imaged on ChemiDoc Imaging System (Bio-Rad, Hercules, CA, USA). The protein panels shown in the figures are a composite of different blots and are representative blots for the indicated proteins and the loading control. Densitometric analysis was performed using ImageJ software (https://imagej.nih.gov/ij/ accessed on 1 May 2022).

### 4.4. Real-Time Cell Analyzer (RTCA) Analysis

The real-time cell analyzer was used to analyze the cell index of untreated and TCDD treated A2780 and A2780cis cells. Briefly, 50,000 cells were seeded in electrodes containing E-Plate 16 as reported before [34]. After 24 h of incubation, cells were treated with varying doses of TCDD and were monitored in real-time for 120 h. The impedance is measured by electronic sensors and expressed as the cell index value.

### 4.5. Clonogenic Assay

Approximately 4000 cells/well seeded in 12-well plates, with and without varying concentrations of TCDD, and grown for two weeks for developing colonies. Upon incubation, the wells were first washed gently with PBS followed by fixing using glutaraldehyde (6.0% *v*/*v*) solution. The colonies in each well were then stained with crystal violet (0.5% *w*/*v*) for 1 h. The colonies were then washed gently in the water, dried, and imaged by a charged-coupled device camera (Nikon D3200).

### 4.6. Apoptosis Assay

The presence of apoptotic cells was analyzed using Annexin-V/Propidium Iodide Dual Staining (BD Biosciences, San Jose, CA, USA) as per the manufacturer’s protocol on BD LSRFortessa analyzer (BD Biosciences) as explained previously [35].

### 4.7. Cell Cycle Analysis

A2780 and A2780cis cells untreated and treated with TCDD were trypsinized, washed, and stained with PI/RNAase solution (25 μg/mL), and the cell cycle stages were examined by flow cytometry by BD LSRFortessa analyzer as explained before [36].

### 4.8. H2AX Phosphorylation Assay

A2780 and A2780cis cells untreated and treated with TCDD were fixed, permeabilized, and then stained with 3 μL of pH2AX (pS139)-Alexa Fluor 647 antibody for 30 min. The cells were then washed with FBS and the content and expression of phosphorylated H2AX were determined by the BD LSRFortessa cell analyzer [37].

### 4.9. Wound Healing/Scratch Assay

Approximately 90% of confluent A2780 and A2780cis cells were scratched from one end of the wall to the other using a sterile 200 μL pipette tip. After washing out the detached cells carefully using PBS, the cells were incubated in fresh media for 24 h without and with 10 nM TCDD. Migration of cells was observed at 0 and 24 h using an Evos microscope, and images were captured at 4× magnification. The experiment was performed in triplicate.

### 4.10. Statistical Analysis

Statistical analysis was carried out using Graph Pad Prism 9.0 software. The differences between control and treatment groups were evaluated using one-way ANOVA, and Dunnett’s test. *p* ≤ 0.05 was considered statistically significant. Data are shown as mean ± SD from three different experiments.

## 5. Conclusions

Taken into account, the current study provides the first evidence that the AhR/CYP1A pathway regulates OC progression via the activation of PI3K/AKT, stemness characteristics via the activation of Wnt/β-catenin and NOTCH pathways, and induces metastasis via inducing epithelial to mesenchymal transition (Figure 5). A further mechanistic study involving the AhR inhibitor would further validate the involvement of AhR in regulating carcinogenesis, proliferation, metastasis, and chemoresistance in OC cells.

## Data Availability

Not applicable.

## Supplementary Materials

The following supporting information can be downloaded at: https://www.mdpi.com/article/10.3390/ijms23126395/s1.
