# Supplementary figures and images for: Aryl Hydrocarbon Receptor Promotes Cell Growth, Stemness Like Characteristics, and Metastasis in Human Ovarian Cancer via Activation of PI3K/Akt, β-Catenin, and Epithelial to Mesenchymal Transition Pathways
Source: Int J Mol Sci. 2022 Jun 7;23(12):6395. doi: 10.3390/ijms23126395 (PMC9223661; doi:10.3390/ijms23126395)

**Figure S1**

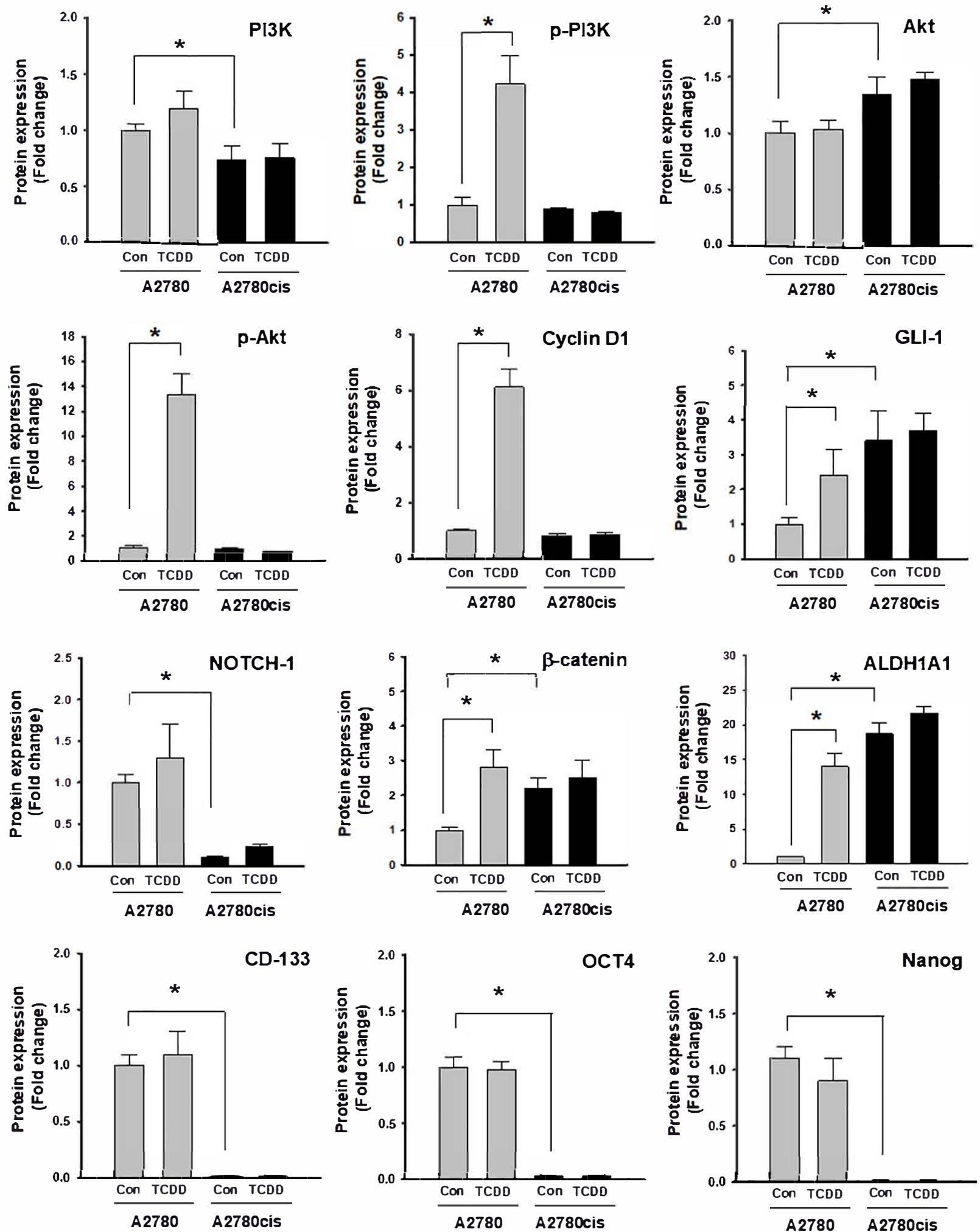

Figure S2

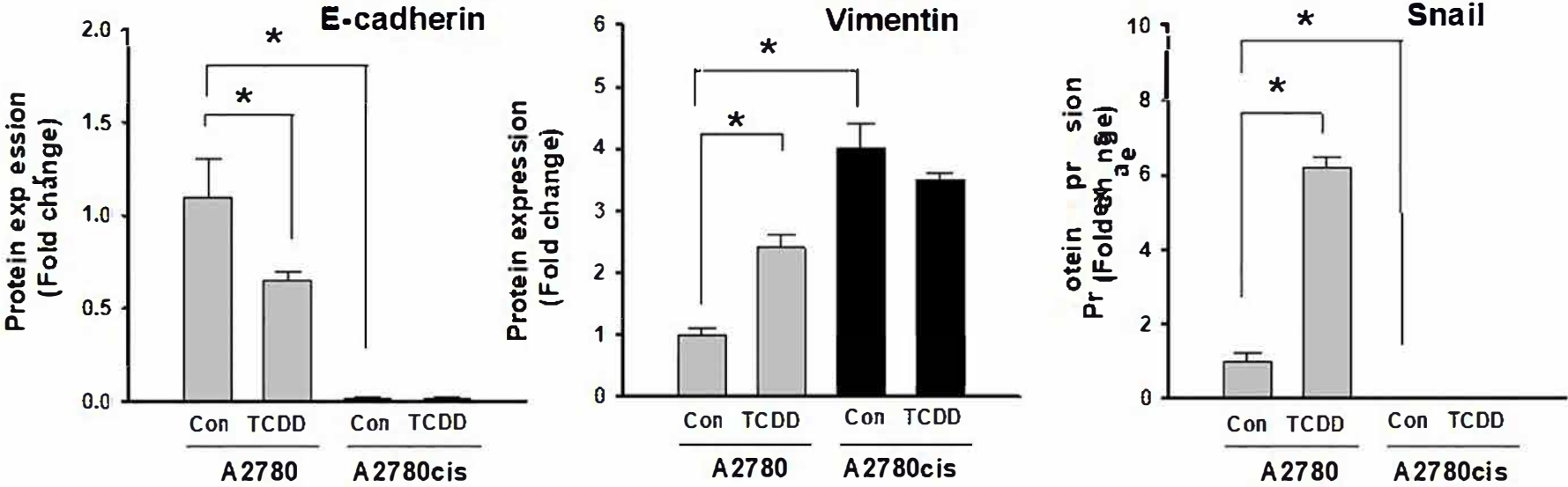

Supplement: Supplementary file 1 [file ijms-23-06395-s001.zip › ijms-1718256-supplementary.pdf]
